# Vegetarians and different types of meat eaters among the Finnish adult population from 2007 to 2017

**DOI:** 10.1017/S0007114521001719

**Published:** 2022-04-14

**Authors:** Elviira Lehto, Niina E. Kaartinen, Katri Sääksjärvi, Satu Männistö, Piia Jallinoja

**Affiliations:** 1 Faculty of Social Sciences, 33014 Tampere University, Tampere, Finland; 2 Finnish Institute for Health and Welfare, Department of Public Health Solutions, 30, 00271 Helsinki, Finland

**Keywords:** Vegetarian, Red and processed meat, Food consumption, Food choice motives, Health behaviour, Lifestyle factors

## Abstract

From health and sustainability perspectives, reduction in the consumption of animal-based foods, especially red meat, is a key strategy. The present study examined the prevalence, sociodemographic and lifestyle factors, food consumption and food choice motives of vegetarians and consumers of low and high amounts of red and processed meat (RPM) among Finnish adults. We applied the data from three national health studies: FINRISK 2007 (*n* 4874), FINRISK 2012 (*n* 4812) and FinHealth 2017 (*n* 4442). Participants addressed their food consumption with a FFQ and answered other questionnaires about sociodemographic and lifestyle factors, as well as food choice motives. The prevalence of vegetarianism increased from 0·7 % in 2012 to 1·8 % in 2017, and median daily RPM consumption decreased from 128 g in 2007 to 119 g in 2012 and to 96 g in 2017. Vegetarians and members of the low-RPM group were more often women, younger and more highly educated than the high-RPM group, both in 2007 and 2017. Still, the importance of sex for the probability of a vegetarian diet decreased, while its importance for high-RPM consumption increased. Vegetarians consumed more fruit, vegetables, legumes, nuts and seeds than either the low- or high-RPM groups. The high-RPM group had the lowest scores in several aspects of healthy and sustainable diet, healthy food choice motives and healthy lifestyle. Vegetarians and groups differing in their RPM consumption levels might benefit from differing interventions and nutrition information taking into account their other dietary habits, food choice motives and lifestyle factors.

The process termed a ‘meatification of human diet^(1)^ describes increased meat consumption, documented for over half a century^(2,3)^. However, recently, only the consumption of poultry has increased, whereas that of pork and beef has either declined or remained rather stable in Finland^(3)^ and in Europe^(4)^. Still, 79 % of men and 26 % of women in Finland exceed the nutritional recommendation of at most 500 g/week of red and processed meat (hereafter referred to as RPM)^(5,6)^.

While holding a central position in Western diets, meat consumption, especially the consumption of RPM, has also been shown to have several negative effects. As regards health, high-RPM consumption has been associated with higher risk of type 2 diabetes^(7)^, some types of cancer^(8)^, stroke^(9)^ and cardiovascular and all-cause mortality^(10,11)^. From a sustainability perspective, the production of animal-based foods creates a vast environmental burden^(12)^, which is highest for ruminant meat, followed by other meat, dairy and plant-based foods^(13)^. Many plant-based foods with the lowest environmental burden are also foods that have been associated with low relative risk of disease or mortality^(14)^. Hence, to address both sustainability and healthy nutrition goals, the EAT-Lancet Commission has stressed the need to reduce consumption of animal-based foods^(15)^.

During recent years, different forms of plant-based diets have been proposed as solutions to the above-mentioned health and sustainability problems, both among the academic community^(13,15)^ and in society and the media more broadly. While plant-based diets such as veganism have turned into trendy lifestyle choices^(16)^, various compromised solutions for reducing meat consumption, such as flexitarianism^(17)^ and ‘Meatless/Meat-Free Mondays’^(18)^, have also been introduced. In Finland, from about 2013 to 2016, several events and occasions – Meatless October Pledge and Veganuary Pledge, to name two – marked a phenomenon that has been termed a ‘veggie boom’^(19)^. Despite this increased popularisation, however, the prevalence of plant-based diets has remained rather low in Western societies, including Finland^(20)^. In 2016, of 15–79-year-old Finns, 1·1 % reported being vegans, 2·5 % vegetarians and 8·3 % following a diet excluding red meat^(21)^.

Previous studies suggest that plant-based diets are not equally popular throughout the whole population. Vegetarians have been more often women, younger and more highly educated than meat eaters^(22,23)^. A UK study^(23)^ also suggested that vegetarians have lower BMI and body fat percentage compared with regular meat eaters, those eating low amounts of meat or poultry vegetarians. In Canada, smoking was less common among vegetarians than among omnivores, whereas other health behaviour differences were highly sex-specific^(24)^. On the other hand, vegans have been found to be less educated than meat eaters^(22)^ and to eat healthier (higher fruit, vegetable and nut consumption) but not to differ from meat eaters in BMI nor in health behaviours such as self-reported physical activity, smoking or alcohol consumption^(25)^. The lack of a clear pattern in the variation of background factors as well as health behaviours among individuals with different levels of meat consumption or among different types of vegetarians might reflect different values and motivations for a certain lifestyle, of which a diet is only one part.

The present study contributes to the above-mentioned previous studies, by analysing changes in vegetarianism and RPM consumption in a moment, when the public images of plant-based diets started to change into more positive and meat was increasingly associated with health- and environment-related problems. The aim of the present study is to explore vegetarian diet and RPM consumption patterns in the Finnish adult population. First, we analyse the prevalence of a vegetarian diet and RPM consumption levels in 2007, 2012 and 2017. Second, we examine the background factors of vegetarians and low- and high-RPM consumption groups and study whether these determinants differ between 2007 and 2017. Finally, we compare the vegetarians, low-RPM consumption group, and high-RPM consumption group in 2017 as regards their consumption of other selected foods, food choice motives and lifestyle factors.

## Methods

### Participants and procedure

**Fig. 1.**
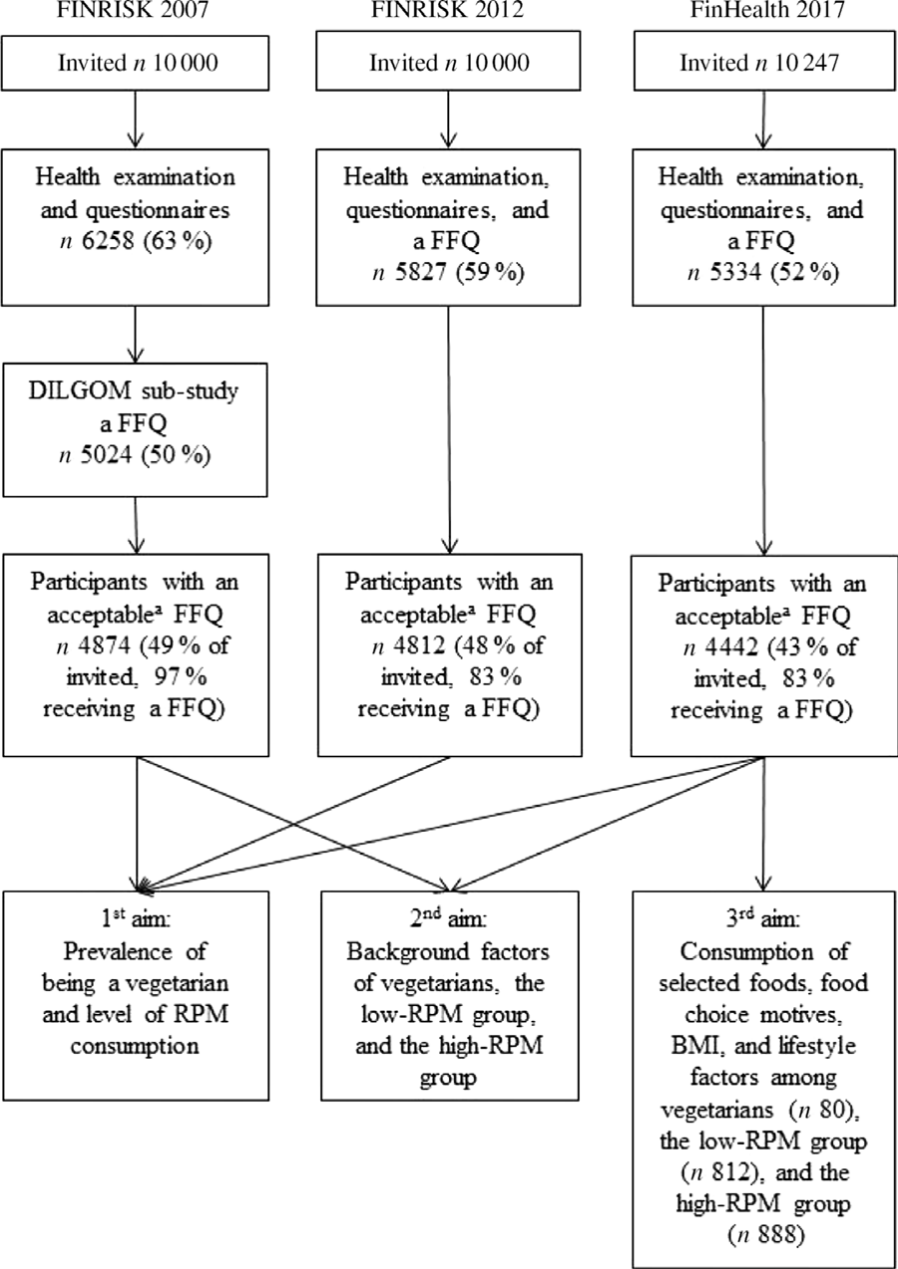
Flow diagram of the participation in FINRISK 2007, FINRISK 2012 and FinHealth 2017 Studies and the aims of the present research. RPM, red and processed meat.

The present research applies the data from three national, population-based, cross-sectional health examination studies: FINRISK 2007, FINRISK 2012 and FinHealth 2017. The FINRISK Studies, described in detail elsewhere^(26)^, were conducted in Finland every 5 years between 1972 and 2012 to monitor chronic disease risk factors. The FINRISK 2007 and 2012 studies comprised representative random samples of 10 000 adults aged 25–74 years from five large geographical areas (North Karelia, Northern Savo, Turku and Loimaa area, Helsinki and Vantaa, and Northern Ostrobothnia) stratified by area, sex and 10-year age group. The FinHealth 2017 Study merged the study methodology and study population recruitment of FINRISK Studies^(26)^ and the Health 2000 Survey^(27)^. Similar to the Health 2000 Survey, the FinHealth 2017 Study drew a nationally representative sample of the Finnish adult population aged 18 years and older (*n* 10 247). Sampling strategy and research methodology of the FinHealth 2017 Study have been described in more detail elsewhere^(28)^. In each study year, the subjects were invited to a health examination including physical measurements and blood samples. The participation rates (health examination) were 63 % (*n* 6258) in 2007, 59 % (*n* 5827) in 2012 and 52 % (*n* 5334) in 2017 (see Fig. 1 for participation with respect to research aims). The questionnaires inquiring health status and health behaviour were provided at the study site, and the participants filled them in during the health examination or later at home. When filled in at home, the participants were asked to mail them back to the institute using provided prepaid envelopes. In FinHealth 2017, the option to fill in questionnaires in electronic format was available. In 2012 and 2017, a FFQ was administered to all health examination participants. In 2007 instead, all FINRISK 2007 participants were invited to a second study phase (the DILGOM Study) conducted during the next 3 months (84 % participation rate, *n* 5024)^(29)^. In this phase, participants filled in an FFQ at the study site.

To be able to compare participants of different study years, we included only those FinHealth 2017 subjects who were 25–74 years old. Furthermore, we restricted our analytical sample to those participants who had filled in the FFQ acceptably. Exclusions were made due to missing or incompletely filled FFQ and implausibly low or high daily energy intake corresponding to 0·5 % at both ends of the energy intake distributions for men and women. Therefore, the final analytical sample sizes for the present study were 4874 (FINRISK 2007), 4812 (FINRISK 2012) and 4442 (FinHealth 2017).

All studies were approved by the Ethics Committee of the Helsinki and Uusimaa University Hospital District. All surveys were conducted in accordance with the Declaration of Helsinki, and all participants provided their written informed consent prior to commencement of the surveys.

### Measures

#### Dietary variables

Information regarding special diets and food consumption is derived from the semi-quantitative, self-administered FFQ (131 items in 2007 and 2012, 134 items in 2017), which measured the habitual food consumption of the previous 12 months, filled in the spring/summer time. This FFQ measures the whole diet and has been continuously updated based on the most recent national dietary surveys and is repeatedly validated to measure the diet of the general Finnish adult population for epidemiological study purposes^(6,30-32)^. The participants indicated how often they ate a certain food, the answer options ranging from never or seldom to six or more times/d. For each of the different food items, the portion size was sex-specific and predefined based on the most recent national dietary surveys^(28)^. To calculate the average daily consumption of each food in grams, alcohol consumption (ethanol intake in g/d), as well as the daily energy intake, we used calculation software developed by the Finnish Institute for Health and Welfare, which utilises the Finnish national food composition database Fineli® (http://www.fineli.fi/)^(33)^. During the calculation of food consumption, all dishes were decomposed to their basic ingredients. Therefore, the estimated consumption of RPM (g/d), for example, included both RPM eaten by itself and that used as an ingredient in other dishes.

##### Definition of vegetarians

The classification of participants as either vegetarians or non-vegetarians was conducted in three steps. First, we used the question ‘Do you follow a special diet?’ with several options, including ‘Vegan (excludes all animal-based ingredients)’ (in 2017) and ‘Vegetarian (includes milk products or eggs)’ (in 2007, 2012 and 2017). Participants were asked to mark on the list all diets that they follow. In this study, we consider all those who defined themselves as vegan or vegetarian to be vegetarians. Second, we excluded those self-reported vegetarians who had also chosen the option ‘Some other diet’ and specified that this diet includes some kind of RPM, poultry or fish (2007, *n* 6; 2012, *n* 9; 2017, *n* 12).

Third, we classified self-reported vegetarians who nevertheless showed consumption of RPM, poultry or fish equal to or greater than 100 g/d in the FFQ as non-vegetarians (2007, *n* 6; 2012, *n* 17; 2017, *n* 10). We chose this cut-off point for the following reason: The FFQ tool aims to measure the relative intake of foods among Finnish adults and uses general food recipes from the Fineli® database for that purpose. Consequently, almost everyone ends up having some amount of RPM, poultry or fish calculated in their diet, including vegetarians, who in the reality might have consumed an equivalent vegetarian dish not included in the database. Hence, vegetarians who consumed <100 g/d of RPM, poultry or fish according to the general recipes were still considered vegetarians. Moreover, a stricter cut-off point (50 g/d of RPM, poultry or fish) would have made the vegetarian group in 2007 too small and prevented comparisons throughout the study years. The interpretations of the results in our additional analyses were very similar when relying only on the self-defined vegetarian status, using a cut-off point of 100 g/d, or using a cut-off point of 50 g/d for the vegetarian group (for comparisons, see Appendices 1–5 presenting the results for self-defined vegetarians and vegetarians with a cut-off point of <50 g/d). Thus, to increase the accuracy of the definition of vegetarians as much as possible, we decided to use the cut-off point of 100 g/d of RPM, poultry or fish for the vegetarian group. In the studies, the median RPM, poultry or fish consumption among vegetarians ranged between 8–11 g/d, 1 g/d and 12–18 g/d, respectively.

##### Red and processed meat consumption

To define RPM consumption, we added together consumption of beef, pork, game, lamb, offal, sausages and other meat products (e.g. meat cuts and sausage cuts) reported in the FFQ. To define the low- and high-RPM groups, participants were categorised into quintiles according to their RPM consumption. The cut-off points for the lowest quintile of RPM consumption were 76 g/d in 2007 and 54 g/d in 2017. If the participant had reported being a vegetarian, they were excluded from the low-RPM group. For the highest quintile of RPM consumption, the cut-off points were 210 g/d in 2007 and 160 g/d in 2017.

##### Consumption of other foods

Poultry consumption includes chicken and turkey. Fish consumption is the total consumption of fish, fish products and shellfish. Egg consumption includes boiled and fried eggs and omelettes. Liquid dairy consumption consists of the consumption of milk, sour milk products and cream. Cheese consumption covers different types of cheese. Consumption of fruit (including berries) and consumption of vegetables (FV) were added up to form the total FV consumption. Consumption of peas, beans, lentils, soya products and other legume products was included in the total legume consumption. Consumption of butter and butter-based fat spreads was inquired about with one item. Consumption of various vegetable margarine products (with 30, 38, 40, 60 or 70 % fat) and consumption of oil were totalled to obtain vegetable fat and oil consumption. Consumption of nuts and seeds was covered by one item. Cereal consumption includes the intake of wheat, rye, oat, barley, rice and other cereals. Consumption of both sweets and chocolate was asked about separately and added together. Consumption of sugary beverages includes the consumption of sugary drinks, sugary cola drinks and juice drinks (excluding 100 % fruit juices).

#### Food choice motives

Food choice motives were inquired about with eight statements, with possible answers including (1) not at all important; (2) not very important; (3) undecided; (4) somewhat important and (5) very important. The statements were ‘It is important to me (a) that my diet contains a lot of meat products, (b) that the food is low in additives, (c) to choose products with low-fat content, (d) to favor food products high in fiber, (e) to avoid food products high in salt, (f) to follow a low-carb diet, g) to eat a lot of vegetables, fruit, and berries, and (h) that food comforts me when I’m sad or stressed.’ Variables were dichotomised so that the two answer options indicating the greatest importance of the food choice motives were added together and compared with answer options representing lesser importance.

#### Background variables

The sex and age of the participants were obtained from the Population Register Center.

Education was asked about in the questionnaire with the question ‘How many years have you gone to school and studied full-time?’ and reported in years. The education levels of each birth year were divided into tertiles and then added together to form groups of low, middle and high education levels. This was done in order to keep the education level comparable among participants of different ages, since the general education level in Finland has increased over the decades.

Relative household income was calculated from the previous year’s gross household income level, which was weighted according to the number of members of the household. Participants chose one option from the predefined income groups. In 2007, the nine income groups ranged from below 10 000 to over 80 000 euros per year. In the years 2012 and 2017, the ten income groups ranged from below 15 000 to over 90 000 euros per year.

#### BMI and lifestyle factors

BMI was calculated by dividing participants’ weight by the square of their height (kg/m^2^). During the health examinations, trained study personnel measured the weight and height of the participants, according to the standard protocols^(34)^. Those who reported being pregnant (2007, *n* 24; 2012, *n* 36; 2017, *n* 28) were excluded from analyses where BMI was used.

Leisure-time physical activity was asked about with the question ‘How much do you exercise and stress yourself physically in your leisure time?’. The answer options were (1) In my leisure time, I read, watch TV and work in the household on tasks that do not make me move much and that do not physically tax me; (2) In my spare time, I walk, cycle or do other exercise at least 4 h/week; (3) In my spare time, I exercise to maintain my physical condition at least 3 h/week and (4) In my spare time, I regularly exercise several times a week by participating in competitive sports or other heavy sports. We categorised the participants as inactive (answer option 1) or active (answer options 2–4). The questions measuring leisure-time physical activity have been found to correlate moderately with accelerometer counts in the working age population^(35)^ and to have good criterion validity against morbidity and mortality among middle-aged Finns^(36)^.

Physical activity due to commuting was assessed using the question ‘How many minutes of walking, biking or other exercise do you do daily while going to work (there and back)?’ The answer options were (1) I do not work or commute to work entirely with a motor vehicle; (2) <15 min/d; (3) 15–29 min/d; (4) 30–44 min/d; (5) 45–59 min/d and (6) more than 1 h/d. Participants who chose answer option 1 were classified as inactive and categories 2–6 were considered active.

Work-related physical activity was assessed using the question ‘How physically demanding is your work?’. Four answer options varied from very light (mostly sitting) to very demanding (e.g. forest and farm work). Participants who chose the first option were considered to be inactive, and all other options were included in the active category.

Smoking was indicated by participants by choosing one of the following options: (1) I have never smoked regularly, (2) I stopped smoking more than half a year ago, (3) I stopped smoking less than half a year ago and (4) I smoke. We created a dichotomous variable classifying participants as regular smokers and those who had never smoked or had quit smoking.

Alcohol consumption was asked about in the FFQ with several items that contributed to alcohol intake (ethanol intake in g/d).

Eating lunch in a workplace/school canteen was inquired about with the question ‘Where do you eat lunch on most workdays?’ (1) I do not eat lunch, (2) I eat a bag lunch at my workplace, (3) At home, (4) In a restaurant/bar/fast-food place, (5) In my workplace/school canteen and (6) Somewhere else. We dichotomised the answer options so that all options other than ‘In my workplace/school canteen’ were added together.

We also asked ‘Is it possible for you to eat in a workplace/school canteen?’ The answer options were (1) Yes, (2) No and (3) I do not work/study, which were dichotomised by adding the second and third options together.

### Statistical methods

Cross tabulation with *post hoc* comparisons (Z-test with Bonferroni correction) provided the difference in the proportion of vegetarians as well as categorical background variables in 2007, 2012 and 2017. Differences across the study years in the mean age and education level were analysed with one-way ANOVA with Bonferroni corrections. We applied Kruskal–Wallis H test to analyse the differences in the median relative household income, RPM consumption and alcohol consumption in 2007, 2012 and 2017. We examined the differences in background factors between vegetarians, the low-RPM group and the high-RPM group in 2007 and 2017 by using logistic regression analyses. In the first analysis, vegetarians were compared with non-vegetarians; in the second analysis, the low-RPM group was compared with other meat eaters (excluding vegetarians) and in the third analyses, the high-RPM group was compared with other meat eaters (excluding vegetarians). These analyses were conducted separately for 2007 and for 2017. All models included as independent variables sex, age, education level and relative household income. To test the changes in these characteristics among vegetarians, the low-RPM group and the high-RPM group between the years 2007 and 2017, we included the year as an interaction term in the analysis. To compare food consumption, food choice motives, BMI and lifestyle factors among vegetarians, the low-RPM group, and the high-RPM group in 2017, we applied ANCOVA (Bonferroni correction) and cross tabulation with *post hoc* comparisons (Z-test with Bonferroni correction). Analyses of food consumption were adjusted for daily energy intake. We also conducted the same analyses with additional adjustments for sex, age, education level and relative household income. Differences in food consumption between vegetarians, the low-RPM group and the high-RPM group with sex interaction were analysed. Since most of the food consumption variables were skewed, we conducted a logarithm transformation prior to the analyses. To be able to interpret the results in g/d, we also back-transformed the variables and reported the geometric means and their 95 % CI. The level of statistical significance was set at *p* < 0·05. We conducted all analyses with SPSS version 25.

## Results

### Participant characteristics

The descriptive characteristics of the participants in each study year are presented in Table 1. The proportion of vegetarians was 0·7 % in the years 2007 and 2012 and 1·8 % in the year 2017 (*P* < 0·001, difference between 2007/2012 and 2017). The proportion of vegetarians as measured by the self-reported special diet question alone (see the exclusion method in Definition of vegetarians) was somewhat higher: 1·0 % in 2007, 1·3 % in 2012 and 2·3 % in 2017 (data not shown). Participants were asked separately whether they follow a vegan diet only in 2017, when 0·2 % (*n* 8) of the participants chose this option. The median consumption of 96 g RPM per d in 2017 was lower compared with 119 g/d in 2007 and 128 g/d in 2012 (*P* < 0·001).

**Table 1.** Characteristics of FINRISK 2007, FINRISK 2012, and FinHealth 2017 Study subjects who completed the FFQ (Numbers and percentages; median and interquartile range (IQR))

	2007 (*n* 4874)		2012 (*n* 4812)		2017 (*n* 4442)	
Sex, women (%)	53·9		54·6		55·4	
Age (years)						
Mean	52		52		53	
sd	13		14*		14*	
Age groups, years (%)						
25–34	12·7		14·2		13·3	
35–44	17·0		17·3		17·0	
45–54	21·7†		20·2		18·9†	
55–64	24·0		22·8		24·4	
65–74	24·6		25·6		26·3	
Education (years)						
Mean	13		13		14	
sd	4‡,†		4‡		4†	
Education level§(%)						
Low	29·5‡		32·9‡		31·4	
Middle	34·9		32·9		33·1	
High	35·6		34·2		35·5	
	Median	IQR_1_, IQR_3_	Median	IQR_1_, IQR_3_	Median	IQR_1_, IQR_3_
Relative household income (k€/year)	23	15, 33‡,†	27	20, 37‡,*	30	20, 42†,*
1st quintile	10	5, 10‡,†	8	8, 13‡,*	11	8, 14†,*
2nd quintile	17	15, 17‡,†	20	19, 20‡,*	22	20, 27†,*
3rd quintile	23	23, 25‡,†	27	26, 30‡,*	31	30, 32†,*
4th quintile	30	30, 33‡,†	36	32, 37‡,*	40	37, 42†,*
5th quintile	43	37, 50‡,†	50	43, 57‡,*	55	50, 63†,*
BMI (kg/m^2^) (%)						
<25	35·5		37·6*		35·2*	
25–29·9	41·8		38·8		39·2	
≥30	22·6†		23·6		25·6†	
Leisure-time PA: inactive (%)	18·7†		19·8*		22·6†,*	
Commuting PA: inactive (%)	60·7‡,†		64·9‡,*		52·9†,*	
Work-related PA: inactive (%)	55·3‡,†		61·6‡		60·9†	
Smoker|| (%)	17·3		+		15·9	
Alcohol consumption¶ (g/d) (median (IQR_1_, IQR_3_))	4	1, 10†	4	1, 10*	3	1, 9†,*
Vegetarian** (%)	0·7†		0·7*		1·8†,*	
RPM consumption†† (g/d) (median (IQR_1_, IQR_3_))	128	85, 190‡,†	119	79, 174‡,*	96	61, 144†,*
1st quintile	56	38, 67‡,†	51	34, 63‡,*	37	22, 46†,*
2nd quintile	96	86, 103‡,†	87	79, 95‡,*	68	61, 75†,*
3rd quintile	129	121, 139‡,†	119	111, 128‡,*	96	89, 104†,*
4th quintile	175	162, 191‡,†	159	148, 174‡,*	132	121, 144†,*
5th quintile	269	233, 336‡,†	244	216, 305‡,*	204	178, 251†,*

PA, physical activity; RPM, red and processed meat.

* Statistically significant difference at *P* < 0·05 between 2012 and 2017.

† Statistically significant difference at *P* < 0·05 between 2007 and 2017.

‡ Statistically significant difference at *P* < 0·05 between 2007 and 2012.

§ Education level was categorised into tertiles according to birth years.

|| Regular smoker *v.* non-smoker/ex-smoker.

¶ Measured as ethanol.

** The group ‘vegetarians’ also includes possible vegans (before 2017) and vegans (2017).

†† The cut-off points for the lowest quintile were 76 (g/d) in 2007 and 54 (g/d) in 2017. The cut-off points for the highest quintile were 210 (g/d) in 2007 and 160 (g/d) in 2017.

#### Vegetarians

Following a vegetarian diet was more common among women than men, both in 2007 and 2017 (Table 2). In 2007, being vegetarian was more common among 25–34-year-olds, who served as a reference group, than among all older age groups. In 2017, being vegetarian was more common among 25–34-year-olds than among those 45 years and older. Having a high education level, compared with the low level of education, increased the likelihood of being vegetarian, but no difference was seen between the middle and low education level in either 2007 or 2017. Those with higher relative household income (fourth and fifth quintile) were less likely to be vegetarians than those in the lowest income quintile in 2007. In 2017, relative household income was not associated with the likelihood of being vegetarian.

**Table 2.** Being in the groups of vegetarians, low red and processed meat (RPM) consumption, or high-RPM consumption in the FINRISK 2007 (*n* 4874) and FinHealth 2017 (*n* 4442) Studies† (Odd ratio and 95 % confidence intervals)

	Vegetarians				Low-RPM‡ group				High-RPM§ group			
	2007 (*n* 35)		2017 (*n* 80)		2007 (*n* 898)		2017 (*n* 812)		2007 (*n* 914)		2017 (*n* 888)	
	OR	95 % CI	OR	95 % CI	OR	95 % CI	OR	95 % CI	OR	95 % CI	OR	95 % CI
Sex												
Men	ref.		ref.		ref.		ref.		ref.		ref.	
Women	8·59	2·61, 28·26***	2·34	1·40, 3·92**,||	3·96	3·33, 4·69***	3·39	3·82, 5·06***	0·18	0·16, 0·22***	0·14	0·11, 0·17***,||
Age group (years)												
25–34	ref.		ref.		ref.		ref.		ref.		ref.	
35–44	0·25	0·08, 0·76*	0·80	0·42, 1·49	0·74	0·55, 0·99*	0·68	0·53, 0·87**	1·51	1·13, 2·02**	0·99	0·69, 1·43
45–54	0·39	0·16, 0·94*	0·44	0·21, 0·91*	0·82	0·70, 1·20	0·62	0·48, 0·78***	1·41	1·07, 1·86*	1·29	0·91, 1·83
55–64	0·19	0·06, 0·60**	0·41	0·20, 0·81*	1·41	1·09, 1·83*	0·95	0·76, 1·19	1·31	0·99, 1·73	1·17	0·84, 1·64
65–74	0·12	0·03, 0·43**	0·28	0·13, 0·58**	1·65	1·27, 2·13**	1·32	1·06, 1·65*	0·92	0·69, 1·22	0·67	0·47, 0·96*
Education level												
Low	ref.		ref.		ref.		ref.		ref.		ref.	
Middle	3·55	0·97, 13·02	1·15	0·60, 2·22	1·31	1·08, 1·60**	1·04	0·87, 1·23	0·87	0·72, 1·05	0·86	0·68, 1·09
High	7·55	2·21, 25·83**	2·54	1·41, 4·56**	1·42	1·16, 1·74**	1·40	1·18, 1·66***	0·70	0·57, 0·85***	0·58	0·45, 0·74***
Relative Household Income (€/year)												
1st quintile	ref.		ref.		ref.		ref.		ref.		ref.	
2nd quintile	0·44	0·15, 1·28	0·96	0·51, 1·83	0·92	0·72, 1·17	0·92	0·74, 1·13	0·84	0·65, 1·07	0·97	0·71, 1·31
3rd quintile	0·49	0·20, 1·24	0·57	0·25, 1·30	0·90	0·71, 1·14	0·89	0·70, 1·12	0·83	0·65, 1·06	0·98	0·71, 1·36
4th quintile	0·21	0·06, 0·76*	0·83	0·42, 1·62	0·88	0·68, 1·14	1·04	0·84, 1·30	0·92	0·71, 1·18	0·72	0·51, 0·99*
5th quintile	0·33	0·12, 0·93*	0·47	0·21, 1·02	0·80	0·62, 1·03	1·03	0·82, 1·30	0·71	0·55, 0·92*	0·78	0·56, 1·09

Statistically significant at level *P < 0·05; **P < 0·01; ***P < 0·001.

† Separate analyses for 2007 and for 2017 were conducted so that vegetarians were compared with non-vegetarians (i.e. meat eaters), the low-RPM group was compared with other meat eaters (excluding vegetarians), and the high-RPM group was compared with other meat eaters (excluding vegetarians). All analyses include the following variables simultaneously in the model: sex (men/women), age group, education level group and relative household income group.

‡ The lowest RPM consumption quintile in the year 2007 (cut-off point 76 g/d) and in 2017 (cut-off point 54 g/d), excluding vegetarians.

§ The highest RPM consumption quintile in the year 2007 (cut-off point 210 g/d) and in 2017 (cut-off point 160 g/d).

|| Statistically significant change between 2007 and 2017 in the importance of the independent variable for the dependent variable.

The effect of sex on being vegetarian decreased from 2007 to 2017, meaning that a higher proportion of men were vegetarians in 2017 than in 2007 (Δ OR 0·27; 95 % CI 0·07, 0·98, *P* < 0·05). Between 2007 and 2017, no other changes were found in the importance of the background factors on the likelihood of being vegetarian.

#### Low-red and processed meat group

Being in the low-RPM group was more common among women than men in both 2007 and 2017 (Table 2). In 2007, 35–44-year-olds were less often in the low-RPM group compared with those aged 25–34 years, whereas 55–74-year-olds were more often in the low-RPM group. In 2017, the pattern was similar, with the addition that 45–54-year-olds also had a lower likelihood of being in the low-RPM group, while only 65–74-year-olds had a higher likelihood of being in the low-RPM group. Being in the middle (2007) or high (2007, 2017) education level group increased the probability of being in the low-RPM group. Income level was not associated with the probability of being in the low-RPM group in 2007 or 2017.

There was no interaction with time, meaning that the effect of the background factors on the likelihood of being in the low-RPM group did not change between 2007 and 2017.

#### High-red and processed meat group

Being in the high-RPM group was more common among men in both 2007 and 2017 (Table 2). In 2007, participants aged 35–54 years were more likely to be in the high-RPM group compared with 25–34-year-olds. In 2017, only the age group of 65–74-year-olds differed from the reference group by having a lower likelihood of being in the high-RPM group. Those in the highest quintile for household relative income (2007) or in the second highest income quintile (2017) had a lower probability of being in the high-RPM group when compared with the lowest income quintile.

The probability of being in the high-RPM group became more sex-dependent: in 2017, women were less likely to be in the group than they were in 2007 (Δ OR 0·73; 95 % CI 0·53, 0·98, *P* < 0·05). Between 2007 and 2017, no other changes were found in the importance of the background factors on being in the high-RPM group.

### Food consumption among vegetarians, the low-red and processed meat group and the high-red and processed meat group

Table 3 presents the consumption of selected foods (in g/d) adjusted for daily energy intake among vegetarians, the low-RPM group and the high-RPM group. By definition, vegetarians consumed less RPM, poultry and fish than the low- or high-RPM groups. Vegetarians consumed the following foods more than both the low- and high-RPM groups: FV, legumes, nuts and seeds, and sweets and chocolate. Liquid dairy consumption was lower among vegetarians than among the low-RPM group but approximately as high as among the high-RPM group. Vegetarians consumed fewer eggs and less butter and butter-based fat spreads but more cereals than the high-RPM group, whereas they consumed these foods in the same amounts as the low-RPM group.

**Table 3.** Adjusted means and 95 % CI* for consumption (g/d) of selected foods in the year 2017 in the groups of vegetarians, low red and processed meat (RPM) consumption or high-RPM consumption (Numbers; mean and 95 % confidence intervals)

		Vegetarians (*n* 80)		Low-RPM† group (*n* 812)		High-RPM‡ group (*n* 888)	
	*n*	Mean	95 % CI	Mean	95 % CI	Mean	95 % CI
RPM	1780	11	10, 12§,||	34	32, 35¶,||	202	194, 210¶,§
Poultry**	1780	2	1, 3§,||	22	20, 25¶,||	27	25, 29¶,§
Fish	1780	11	9, 13§,||	41	38, 44¶	39	36, 41¶
Eggs	1780	19	16, 23||	22	21, 24||	29	27, 31¶,§
Liquid dairy products	1780	307	257, 366§	403	378, 429¶,||	305	288, 324§
Cheese	1780	41	34, 49	42	39, 45||	33	31, 35§
Butter and butter-based fat spreads	1780	6	5, 8||	7	7, 8||	9	9, 10¶,§
Vegetable margarine and oil	1780	16	13, 19	16	15, 17||	14	13, 15§
FV	1780	592	514, 681§,||	446	425, 468¶,||	307	292, 322¶,§
Legumes	1780	41	34, 50§,||	10	10, 11¶	10	9, 10¶
Nuts and seeds	1780	10	8, 13§,||	6	5, 6¶,||	2	2, 2¶,§
Cereal products	1780	119	109, 131||	123	119, 127||	103	100, 106¶,§
Rye††	1772	29	23, 37	31	29, 34||	25	23, 27§
Sweets and chocolate	1780	15	12, 18§,||	9	9, 10¶	9	8, 10¶
Sugary beverages	1775	29	20, 40	21	19, 24||	37	33, 42§

FV, fruit and vegetables; RPM, red and processed meat.

* Geometric means and 95 % CI for LG10 transformed and back-transformed food consumption variables, which were analysed with ANCOVA (Bonferroni corrections), adjusted for daily energy intake.

† The lowest red and processed meat consumption quintile in the year 2017: cut-off point 54 g/d, excluding vegetarians.

‡ The highest red and processed meat consumption quintile in the year 2017: cut-off point 160 g/d.

§ Statistically significant difference at level *P* < 0·05 with the low-RPM group.

|| Statistically significant difference at level *P* < 0·05 with the high-RPM group.

¶ Statistically significant difference at level *P* < 0·05 with vegetarians.

** Sex interaction.

†† Rye is the most important determinant of whole-grain intake in Finland.

In addition to RPM, the high-RPM group also consumed more poultry, eggs, butter and butter-based fat spreads, and sugary beverages, but less liquid dairy, cheese, vegetable fats and oil, FV, nuts and seeds, and cereals, than the low-RPM group. Members of the low- and high-RPM groups did not differ from each other in their consumption of fish, legumes, and sweets and chocolate.

We ran the same analyses examining food consumption in the three groups with additional adjustments for sex, age, education level and relative household income, but the results remained essentially unchanged (Appendix 6). However, the difference in poultry consumption between the low- and high-RPM groups and the difference in consumption of sweets and chocolate between vegetarians and the high-RPM group became non-significant after these adjustments.

Interaction with sex was found with poultry consumption (data not shown). While men in the high-RPM group had higher poultry consumption than those in the low-RPM group, poultry consumption among women was equal among both RPM consumption groups.

### Food choice motives among vegetarians, the low-red and processed meat group and the high-red and processed meat group

As expected, none of the vegetarians reported that they consider it important that their food is high in meat, thus differing from both the low- and high-RPM consumption groups (Table 4). On the contrary, all vegetarians considered it important that their food is high in FV. High meat content was also less important, and high FV content more important, for the low-RPM group, compared with the high-RPM group. Still, 78·4 % of those in the high-RPM group considered FV important in their diet. Both vegetarians and members of the low-RPM group were more likely to value a lack of additives and high level of fibre in their food, compared with the high-RPM group. Low salt content and low level of fat in food were more important for the low-RPM group, compared with the high-RPM group. Low levels of carbohydrates or comfort from food when sad or depressed were less important food choice motives among all participants, although vegetarians valued the comforting nature of food more often than the low- or high-RPM groups.

**Table 4.** Percentages of food choice motives, BMI and lifestyle factors and adjusted mean and 95 % CI* for alcohol consumption (g/d) in the year 2017 in the groups of vegetarians, low red and processed meat (RPM) consumption, or high-RPM consumption (Number and percentages; mean and 95 % confidence intervals)

		Vegetarians (*n* 80)		Low-RPM† group (*n* 812)		High-RPM‡ group (*n* 888)	
	*n*	%		%		%	
Food choice motives: Important that food…							
is high in meat	1746	0·0§,||		13·6¶,||		62·1¶,§	
is high in FV	1749	100·0§,||		90·7¶,||		78·4¶,§	
is low in fat	1748	62·5		68·5||		50·3§	
is high in fibre	1747	84·8||		84·1||		64·5¶,§	
is low in salt	1753	66·3		75·6||		59·5§	
is low in carbohydrates	1744	31·3		28·5		23·2	
contains no additives	1748	80·0||		75·9||		59·0¶,§	
comforts when sad or stressed	1752	28·7§,||		15·6¶		15·6¶	
Possibility to eat in workplace/school canteen	1757	37·7		32·0		35·3	
Eats lunch in workplace/school canteen	1769	22·8		13·9||		18·5§	
BMI < 25 kg/m^2^	1754	47·4||		44·8||		25·6¶,§	
Leisure-time PA: inactive	1765	24·1		22·2		25·1	
Commuting PA: inactive	1093	28·8||		36·9||		63·6¶,§	
Work-related PA: inactive	1745	59·7		68·0||		55·7§	
Smoking regularly	1759	11·3		13·2||		20·5§	
	*n*	Mean	95 % CI	Mean	95 % CI	Mean	95 % CI
Alcohol consumption**,††	1780	2	2, 3||	3	2, 3||	5	5, 6¶,§

PA, physical activity; RPM, red and processed meat.

* Geometric mean and 95 % CI for LG10 transformed and back-transformed alcohol consumption variable, which was analysed with ANCOVA (Bonferroni corrections), adjusted for daily energy intake. All other variables were analysed unadjusted (*χ*^2^ test).

† The lowest red and processed meat consumption quintile in the year 2017: cut-off point 54 g/d, excluding vegetarians.

‡ The highest red and processed meat consumption quintile in the year 2017: cut-off point 160 g/d.

§ Statistically significant difference at level *P* < 0·05 with the low-RPM group.

|| Statistically significant difference at level *P* < 0·05 with the high-RPM group.

¶ Statistically significant difference at level *P* < 0·05 with vegetarians.

** Measured as ethanol g/d.

†† Sex interaction.

### BMI and lifestyle factors among vegetarians, the low-red and processed meat group and the high-red and processed meat group

As regards the possibility of eating in a workplace/school canteen or actually eating there, BMI, physical activity, smoking and alcohol consumption, vegetarians did not differ from the low-RPM group (Table 4). Vegetarians did, however, more often have a BMI below 25 kg/m^2^, were more often physically active during their work commutes and consumed less alcohol than those in the high-RPM group. Regular smoking was also borderline significantly less common among vegetarians than among the high-RPM group (*P* = 0·051). High-RPM group members, compared with the low-RPM group, more often ate lunch in a workplace/school canteen, had a BMI higher than 25 kg/m^2^ and were physically inactive when commuting to work, but they were less likely to be physically inactive in their work. In addition, high-RPM group members were more often smokers and consumed more alcohol than low-RPM group members.

Interaction with sex was found in alcohol consumption (data not shown): men in the high-RPM group consumed more alcohol than those in the low-RPM group, while among women, no differences were found between the low- and high-RPM groups.

## Discussion

The present study conducted in the Finnish adult population showed an increase in the prevalence of vegetarians and decrease in RPM consumption between 2007 and 2017. In both study years, vegetarians and members of low-RPM group were more often women, younger and more highly educated than the high-RPM group. Between 2007 and 2017, the probability of a vegetarian diet increased especially among men, whereas the probability of belonging to the high-RPM group decreased among women. Vegetarians, compared with low-RPM and high-RPM groups, consumed more foods considered healthy and sustainable such as fruit, vegetables, legumes, nuts and seeds. Moreover, those in the high-RPM group had the lowest scores in several aspects of healthy and sustainable diet, healthy food choice motives and healthy lifestyle.

Prior to our research period, between 1997 and 2002, the percentage of vegetarians in the Finnish adult population remained at a level of approximately 0·4 %^(37)^. Our study showed no difference in the prevalence of vegetarians between 2007 and 2012. By 2017, however, the popularity of the vegetarian diet had increased to 1·8 %, with 0·2 % being vegans. Others, too, have reported an increase in self-reported followers of vegan and vegetarian diets and diets excluding red meat between 2014 and 2016^(21)^ and between 2016 and 2018 in Finland^(20)^. The proportion of Finns following a vegetarian diet may seem low, when compared, for example, with the results of Swedish polls stating that the proportion of self-reported vegetarians was 4 % in 2009 and 7 % in 2018^(20,38,39)^. Country comparisons are, however, challenging due to methodological differences, as, in addition to polls, many previously referred studies have applied convenience samples and stated only self-reported special diets without combining them with FFQ indications of dietary choices^(40)^. A recent Swiss study that defined vegetarians based on the information of a FFQ showed somewhat similar moderate results as our study: the prevalence of vegetarians had increased from 0·5 % in 2005–2009 to 1·2 % in 2016–2017^(41)^.

Our study also suggests a decrease in the consumption of RPM between 2007 and 2012 and between 2012 and 2017. This finding is in line with previous reports showing a decline in or stabilisation of the consumption of beef and pork^(3)^ and might partly be a reflection of the ‘veggie boom’ described in previous studies^(16,19,42)^. Since the consumption of poultry has increasingly grown during the past decade^(3)^, however, some part of meat consumption may have shifted from RPM to poultry. Moreover, the results of the current research are in line with the national dietary survey from year 2017 that showed mean usual RPM consumption in Finnish adults (108 g/d in men and 56 g/d in women)^(6)^ to be still far from the daily maximum limit of 14 g beef, lamb or pork suggested by the EAT-Lancet Commission^(15)^.

Being vegetarian was more common among women and younger and more highly educated people in both 2007 and 2017. Several previous studies of Western populations have concluded that a vegetarian diet is more prevalent among women than men^(22,37,40)^. In our study, however, the vegetarian diet became more popular especially among men between 2007 and 2017. The decreasingly gendered nature of vegetarianism may also point to a mainstreaming and normalisation of vegetarian eating^(19)^, although in regard of age, education or income level, no changes were noted.

As regards RPM consumption, women and more highly educated were more often in the low-RPM group and less often in the high-RPM group in both study years. With respect to female sex, younger age and high education level, low-RPM group members resembled vegetarians, but being in the low-RPM group was also more common among those over 65 years. Although higher RPM consumption among men has been noted earlier in Finland and in Baltic countries^(43,44)^, the increased importance of male sex for the probability of high-RPM consumption between 2007 and 2017 was somewhat surprising. It is possible that since women can more often be eager to change their diet^(45)^, the changes in RPM consumption were seen among women only in the high-RPM group, simply because the reduction of RPM is easier when one’s diet contains higher, rather than lower, levels of RPM. This implies that attempts to decrease excessive RPM consumption should target especially men. Additional research is needed to demonstrate the most effective ways to reach men in the high-RPM group and to show whether sex, age or education level differentiate the measures otherwise found to lead in reduced meat consumption^(46)^.

Food consumption among vegetarians included several healthy aspects: they reported lower consumption of butter and butter-based fat spreads than the high-RPM group and higher consumption of FV, legumes, and nuts and seeds, compared with the low- or high-RPM groups. Others, too, have reported somewhat similar results, especially regarding higher FV intake among vegetarians compared with non-vegetarians^(23,24,47)^. Belgian vegetarians scored higher on the Healthy Eating Index 2010 mainly due to their higher consumption of FV, lower consumption of saturated fat and lower Na intake when compared with an omnivorous diet^(48)^. One of the less healthy diet characteristics among vegetarians in our study was their consumption of sweets and chocolate, which was higher than that of low- or high-RPM group members. The difference between vegetarians and the high-RPM group attenuated, however, when we adjusted the analyses for sex, age, education and income.

When comparing only groups with differing levels of RPM consumption, high-RPM consumption has previously been associated with lower consumption of fruits, whole grains and nuts in Finnish adults^(43)^. Similarly, in our study, the high-RPM group reported lower consumption of FV, cereals, and nuts and seeds, but higher consumption of eggs, butter and butter-based fat spreads, and sugary beverages, compared with the low-RPM group. Interestingly, low-RPM group members consumed liquid dairy and cheese even more than high-RPM group members, differing also from vegetarians in their higher consumption of liquid dairy. It is also notable that vegetarians did not differ from high-RPM group members in their consumption of liquid dairy and cheese, which was also relatively high from a sustainability perspective^(15)^. Dairy seems to be an important part of the diet of the Finnish adult population, with vegetarians, low-RPM group members and high-RPM group members differing from each other only in the types of dairy food they consume.

Vegetarians and low-RPM group members resembled each other in most of the food choice motives we analysed here. The most striking differences between the groups were in the importance of meat, FV, food additives and fibre, the high-RPM group favouring meat the most, while favouring FV and fibre and avoiding additives the least. Despite these differences between the groups, however, there were still many similar tendencies. A majority of each study group valued a diet high in FV and fibre and low in additives, with vegetarians and low-RPM group members more often seeing these as important and high-RPM group members least often seeing them as important. Previously, compared with non-vegetarians, vegetarians have more often reported choosing foods based on their nutrient or low-fat content, other health-related food choice motives being more sex-dependent^(24)^. In the present study, however, only low-RPM group members considered low-fat content more important than high-RPM group members did.

It is notable that although a majority of high-RPM group members emphasised the importance of high meat content in their diet, approximately one-third did not consider that to be of utmost importance. This indicates that even those in the highest RPM consumption quintile might not entirely oppose some reduction of the meat content in their diet. In a study of British university canteens^(49)^, for example, increasing the availability of vegetarian options increased the sales of vegetarian dishes especially among those who previously least often chose vegetarian options. Since those in the high-RPM group were equally likely to have the opportunity to eat lunch in a workplace or school canteen, and actually ate there slightly more often than the low-RPM group, offering more plant-based options in canteens could direct the high-RPM group to choose healthier and more sustainable lunch options. At least in some respect, eating in a workplace/school canteen has been associated with a diet closer to Finnish nutrition recommendations both among women and men^(50)^.

As regards BMI and lifestyle factors, vegetarians and the low-RPM group did not differ from each other, but high-RPM group members were more often physically inactive when commuting to work, more likely to be overweight or obese, to be regular smokers and to consume more alcohol. This is somewhat in contrast with a previous UK study that reported vegetarians to be of normal weight more often than either consumers of low amounts of meat or regular meat eaters^(23)^. A Canadian study^(24)^, on the other hand, reported that vegetarians smoke less often than non-vegetarians but that lower BMI and higher PA were more apparent only among vegetarian women and not vegetarian men, compared with non-vegetarians.

Vegetarians have been reported to be more health conscious than meat eaters^(24)^, but most studies reporting health behaviour differences between vegetarians and omnivores have not made a distinction between different levels of RPM consumption in the omnivorous group. In our study, the lack of differences in lifestyle factors between vegetarians and the low-RPM group might relate to female sex and high education level, which defined both groups. Moreover, low-RPM group members seemed to be at least as health-oriented as vegetarians, if not more so. This might be due to the fact that, along with health benefits, vegetarians more often point to the importance of animal rights or ecological concerns as the main motive for their diet^(51)^. Unfortunately, ethical or environmental food choice motives were not inquired in the present study. When studying food consumption and food choice motives, one has to also bear in mind that not all food choices are equally voluntary. Some diseases or conditions such as lactose intolerance as well as financial difficulties might direct the food consumption along with personal will.

Putting together our results in the areas of food consumption, food choice motives and lifestyle factors among the three groups, vegetarians and the low-RPM group resembled each other in their greater orientation towards a healthy lifestyle, which was less common among high-RPM group members. This suggests the clustering of unhealthy behaviours shown in some previous studies^(43,52)^.

Comprehensive population-based sampling is one of the main strengths of our study, although only the FinHealth 2017 sample was nationally representative. Since we did not use sampling weights, our results can be generalized to those participating in health examination surveys, not to the Finnish general population overall. Thus, we should bear in mind the differences in sampling of geographical areas between 2007 and 2017 while interpreting the changes between these years. In addition, the prevalence of vegetarians, and especially vegans, was low, which did not allow us to examine the associations more specifically, by stratifying them by sex or age, for example. Including younger age groups in the data would have enabled more detailed analysis of vegetarians, as this diet seems to be more popular among young women especially^(53,54)^. Given these limitations, our study, with more representative data, provides new insight into three distinct groups defined by their level of RPM consumption in the Finnish adult population in general.

The validation studies of the FFQ have shown acceptable results with regard to macronutrients and proven useful in epidemiological studies in Finnish adults^(30-32)^. When examining the diet of vegetarians, one challenge is the type of foods included in the FFQ. They represent the food most commonly eaten among the general population and are not specific to a vegetarian diet. Therefore, vegetarians appear to have consumed meat when in reality they have eaten an equivalent vegetarian dish. We aimed to ensure the accuracy of our data by excluding self-defined vegetarians who consumed high amounts of RPM, poultry or fish based on their FFQ reports. A lower cut-off point than 100 g/d of RPM, poultry, or fish used in the present study could have yielded in even better accuracy, but due to the small size of the vegetarian group in 2007, it was not possible to make this strict limitation for all analyses we conducted. The results of the additional analyses in which we limited RPM, poultry or fish consumption to <50 g/d among vegetarians in 2017 (Appendices 3 and 5), did not significantly differ from the results for the vegetarian group with a cut-off point of 100 g/d or alter the interpretation of the associations we have presented above. One additional point in favour of the chosen cut-off point is that 25 % of the vegetarians, whose RPM, poultry or fish consumption was 50–100 g/d, had started to be vegetarians within the same or previous year. This means that these vegetarians could have correctly counted and reported their actual average RPM, poultry and fish consumption during the previous 12 months in which they were not vegetarians yet.

Regardless of the above-mentioned measures, the present study suffers from the problematics of defining vegetarians: in most studies, many self-defined vegetarians turn out to be non-vegetarians, meaning that they are found to consume significant amounts of meat, poultry or fish when their actual food consumption is studied^(37,55,56)^. In our study, especially men who defined themselves as vegetarians seemed to have higher RPM and poultry consumption based on the FFQ compared with women in the self-defined vegetarian group (Appendix 2). Otherwise the results of the additional analyses we conducted with self-defined vegetarians (Appendices 1, 2 and 4) did not differ greatly from the results we have reported for vegetarians, whose RPM, poultry or fish consumption was limited to <100 g/d. We consider it, however, by and large important to make the definition of vegetarians as accurate as possible, since more striking differences could be found, for example, if food choice motives related to ethics and sustainability were studied. It is also an interesting question, whether this lax self-definition as vegetarian hinders these people from taking measures to actually eat according to their values – being it health, ethics or sustainability. From the perspective of a healthy and sustainable diet^(15)^, these semi-vegetarians could probably be placed near low-level meat eaters, in the long continuum ranging from strict vegans to excessive meat eaters. Therefore, people following diets with varying levels of animal-based foods and with differing food choice motives could benefit from more specific information on ways of increasing both the healthiness and sustainability of their diet.

## Supplementary material

For supplementary material accompanying this paper visit https://doi.org/10.1017/S0007114521001719. click here to view supplementary material
