# Supplementary material for: Vegetarians and different types of meat eaters among the Finnish adult population from 2007 to 2017
Source: Br J Nutr. 2021 Jun 28;127(7):1060–72. doi: 10.1017/S0007114521001719 (PMC8924490; doi:10.1017/S0007114521001719)
Supplement: Supplementary file 1 [file S0007114521001719sup001.docx]

Appendix 1. Odds ratios (OR) and 95% confidence intervals (CI) for being in the groups of vegetarians (self-defined), low red and processed meat (RPM) consumption, or high RPM consumption in the FINRISK 2007 (n=4874) and FinHealth 2017 (n= 4442) Studies^a^.

|  | Vegetarians | | Low-RPM^b^ group | | High-RPM^c^ group | |
| --- | --- | --- | --- | --- | --- | --- |
|  | 2007 (n=45) | 2017 (n=97) | 2007 (n=890) | 2017 (n=754) | 2007 (n=913) | 2017 (n=844) |
|  | OR (95% CI) | OR (95% CI) | OR (95% CI) | OR (95% CI) | OR (95% CI) | OR (95% CI) |
| Gender |  |  |  |  |  |  |
| Man | ref. | ref. | ref. | ref. | ref. | ref. |
| Woman | **5.53 (2.33-13.15)^***^** | **2.00 (1.28-3.12)^**^**^Δ^ | **4.00 (3.33-4.69)^***^** | **3.82 (3.16-4.61)^***^** | **0.19 (0.16-0.22)^***^** | **0.17 (0.14-0.21)^***^** |
| Age group, years |  |  |  |  |  |  |
| 25-34 | ref. | ref. | ref. | ref. | ref. | ref. |
| 35-44 | **0.19 (0.07-0.58)^**^** | 0.95 (0.54-1.67)^Δ^ | **0.74 (0.55-0.99)^*^** | **0.68 (0.50-0.94)^*^** | **1.49 (1.11-1.99)^**^** | 1.07 (0.79-1.44) |
| 45-54 | **0.38 (0.17-0.84)^*^** | **0.50 (0.26-0.95)^*^** | 0.90 (0.68-1.19) | **0.80 (0.59-1.09)** | **1.39 (1.05-1.84)^*^** | **1.41 (1.06-1.88)^*^** |
| 55-64 | **0.23 (0.09-0.58)^**^** | **0.39 (0.21-0.75)^**^** | **1.39 (1.07-1.81)^*^** | 1.17 (0.89-1.54) | 1.28 (0.97-1.69) | 1.27 (0.96-1.67) |
| 65-74 | **0.23 (0.09-0.55)^**^** | **0.29 (0.14-0.57)^***^** | **1.61 (1.24-2.09)^***^** | **1.48 (1.13-1.94)^**^** | 0.91 (0.68-1.20) | 0.75 (0.56-1.00) |
| Education level |  |  |  |  |  |  |
| Low | ref. | ref. | ref. | ref. | ref. | ref. |
| Middle | **4.08 (1.34-12.19)^*^** | 1.31 (0.73-2.36) | **1.33 (1.09-1.62)^**^** | 1.18 (0.95-1.46) | 0.87 (0.72-1.05) | 0.96 (0.79-1.17) |
| High | **6.63 (2.27-19.38)^***^** | **2.51 (1.46-4.31)^***^** | **1.44 (1.18-1.77)^***^** | **1.69 (1.36-2.10)^***^** | **0.71 (0.58-0.86)^***^** | **0.63 (0.51-0.78)^***^** |
| Relative Household Income (€/year) |  |  |  |  |  |  |
| 1^st^ quintile | ref. | ref. | ref. | ref. | ref. | ref. |
| 2^nd^ quintile | 0.51 (0.20-1.32) | 0.91 (0.50-1.66) | 0.90 (0.71-1.15) | 0.88 (0.69-1.13) | 0.83 (0.65-1.07) | 1.04 (0.81-1.35) |
| 3^rd^ quintile | 0.79 (0.36-1.73) | 0.70 (0.35-1.43) | 0.88 (0.69-1.12) | **0.74 (0.55-0.99)^*^** | 0.83 (0.65-1.06) | 1.20 (0.91-1.57) |
| 4^th^ quintile | **0.28 (0.09-0.83)^*^** | 0.90 (0.49-1.66) | 0.86 (0.66-1.12) | 1.00 (0.77-1.30) | 0.91 (0.71-1.17) | 1.03 (0.79-1.34) |
| 5^th^ quintile | **0.38 (0.14-0.99)^*^** | 0.59 (0.30-1.16) | 0.78 (0.60-1.01) | 0.79 (0.59-1.05) | **0.71 (0.55-0.92)^**^** | 0.96 (0.73-1.26) |

^a^ Separate analyses for 2007 and for 2017 were conducted so that vegetarians were compared to non-vegetarians (i.e. meat-eaters), the low-RPM group was compared to other meat-eaters (excluding vegetarians), and the high-RPM group was compared to other meat-eaters (excluding vegetarians). All analyses include the following variables simultaneously in the model: gender (man/woman), age group, education level group, and relative household income group.

^a^ The lowest RPM consumption quintile in the year 2007 (cutoff point 76 g/day) and in 2017 (cutoff point 54 g/day), excluding vegetarians.

^b^ The highest RPM consumption quintile in the year 2007 (cutoff point 210 g/day) and in 2017 (cutoff point 160 g/day).

Statistically significant at level ^*^p <.05; ^**^p <.01; ^***^p<.001.

^Δ^ Statistically significant change between 2007 and 2017 in the importance of the independent variable for the dependent variable.

Appendix 2. Adjusted means and 95% confidence intervals (CI)^a^ for consumption (g/day) of selected foods in the year 2017 in the groups of vegetarians (self-defined), low red and processed meat (RPM) consumption, or high RPM consumption.

|  |  | Vegetarians (n=98) | Low-RPM^b^ group (n=799) | High-RPM^c^ group (n=888) |
| --- | --- | --- | --- | --- |
|  | n | Mean (95% CI) | Mean (95% CI) | Mean (95% CI) |
| RPM^†^ | 1785 | 12 (11-14)^l,h^ | 34 (33-36)^v,h^ | 200 (192-208)^v,l^ |
| Poultry^†^ | 1785 | 3 (2-4)^l,h^ | 23 (21-25)^v^ | 27 (25-29)^v^ |
| Fish | 1785 | 15 (13-18)^l,h^ | 41 (38-44)^v^ | 39 (36-41)^v^ |
| Eggs | 1785 | 21 (18-25)^h^ | 22 (21-24)^h^ | 29 (27-31)^v,l^ |
| Liquid dairy products | 1785 | 308 (262-361)^l^ | 406 (380-433)^v,h^ | 305 (287-325)^l^ |
| Cheese | 1785 | 41 (35-49) | 42 (39-44)^h^ | 33 (31-35)^l^ |
| Butter and butter-based fat spreads | 1785 | 6 (5-8)^h^ | 8 (7-8)^h^ | 9 (9-10)^v,l^ |
| Vegetable margarine and oil | 1785 | 15 (13-18) | 16 (15-17)^h^ | 14 (13-15)^l^ |
| FV | 1785 | 585 (516-662)^l,h^ | 443 (421-466)^v,h^ | 307 (293-321)^v,l^ |
| Legumes | 1785 | 39 (33-46)^l,h^ | 10 (10-11)^v^ | 10 (9-10)^v^ |
| Nuts and seeds | 1785 | 10 (8-12)^l,h^ | 6 (5-6)^v,h^ | 2 (2-2)^v,l^ |
| Cereal products | 1785 | 117 (108-128)^h^ | 123 (119-128)^h^ | 103 (100-106)^v,l^ |
| Rye^e^ | 1785 | 29 (23-36) | 31 (29-34)^h^ | 25 (23-27)^l^ |
| Sweets and chocolate | 1785 | 15 (12-18)^l,h^ | 9 (8-10)^v^ | 9 (8-10)^v^ |
| Sugary beverages | 1759 | 28 (21-38) | 22 (19-24)^h^ | 37 (33-42)^l^ |

FV, Fruit and vegetables; RPM, Red and processed meat

^a^ Geometric means and 95% CIs for LG10 transformed and back-transformed food consumption variables, which were analyzed with analyses of covariance (Bonferroni corrections), adjusted for daily energy intake.

^b^ The lowest red and processed meat consumption quintile in the year 2017: cutoff point 54 g/day, excluding vegetarians.

^c^ The highest red and processed meat consumption quintile in the year 2017: cutoff point 160 g/day.

^e^ Rye is the most important determinant of whole grain intake in Finland.

^v^ Statistically significant difference at level p <.05 with vegetarians.

^l^ Statistically significant difference at level p <.05 with the low-RPM group.

^h^ Statistically significant difference at level p <.05 with the high-RPM group.

^†^ Gender interaction: mean daily consumption of RPM among self-defined vegetarians (men: 22 g [95% CI: 19-26] vs. women: 11 g [95% CI: 9-12]), in the low-RPM group (men: 39 g [95% CI: 36-43] vs. women: 33 g [95% CI: 31-35]), and in the high-RPM group (men: 218 g [95% CI: 211-224] vs. women: 182 g [95% CI: 165-200]) as well as mean daily consumption of poultry among self-defined vegetarians (men: 8 g [95% CI: 5-12] vs. women: 3 g [95% CI: 2-3]).

Appendix 3. Adjusted means and 95% confidence intervals (CI)^a^ for consumption (g/day) of selected foods in the year 2017 in the groups of vegetarians (cutoff point < 50 g/day of RPM, poultry, or fish), low red and processed meat (RPM) consumption, or high RPM consumption.

|  |  | Vegetarians (n=60) | Low-RPM^b^ group (n=828) | High-RPM^c^ group (n=888) |
| --- | --- | --- | --- | --- |
|  | n | Mean (95% CI) | Mean (95% CI) | Mean (95% CI) |
| RPM | 1776 | 8 (7-10)^l,h^ | 33 (32-34)^v,h^ | 202 (194-210)^v,l^ |
| Poultry | 1776 | 1 (1-2)^l,h^ | 22 (20-24)^v,h^ | 27 (25-30)^v,l^ |
| Fish | 1776 | 7 (5-8)^l,h^ | 41 (38-44)^v^ | 39 (37-42)^v^ |
| Eggs | 1776 | 15 (12-19)^l,h^ | 23 (21-24)^v,h^ | 29 (27-31)^v,l^ |
| Liquid dairy products | 1776 | 282 (230-345)^l^ | 403 (378-429)^v,h^ | 306 (288-326)^l^ |
| Cheese | 1776 | 39 (31-49) | 42 (39-45)^h^ | 33 (31-35)^l^ |
| Butter and butter-based fat spreads | 1776 | 6 (5-8)^h^ | 7 (7-8)^h^ | 9 (9-10)^v,l^ |
| Vegetable margarine and oil | 1776 | 15 (12-18) | 16 (15-17)^h^ | 14 (13-15)^l^ |
| FV | 1776 | 574 (490-673)^l,h^ | 450 (428-473)^v,h^ | 307 (292-322)^v,l^ |
| Legumes | 1776 | 41 (33-51)^l,h^ | 11 (10-11)^v^ | 10 (9-10)^v^ |
| Nuts and seeds | 1776 | 11 (8-15)^l,h^ | 6 (5-6)^v,h^ | 2 (2-2)^v,l^ |
| Cereal products | 1776 | 120 (108-133)^h^ | 122 (119-126)^h^ | 103 (100-106)^v,l^ |
| Rye^e^ | 1776 | 28 (21-38) | 31 (29-34)^h^ | 25 (23-27)^l^ |
| Sweets and chocolate | 1776 | 17 (14-22)^l,h^ | 9 (9-10)^v^ | 9 (8-10)^v^ |
| Sugary beverages | 1751 | 30 (20-43) | 21 (19-24)^h^ | 37 (33-42)^l^ |

FV, Fruit and vegetables; RPM, Red and processed meat

^a^ Geometric means and 95% CIs for LG10 transformed and back-transformed food consumption variables, which were analyzed with analyses of covariance (Bonferroni corrections), adjusted for daily energy intake.

^b^ The lowest red and processed meat consumption quintile in the year 2017: cutoff point 54 g/day, excluding vegetarians.

^c^ The highest red and processed meat consumption quintile in the year 2017: cutoff point 160 g/day.

^e^ Rye is the most important determinant of whole grain intake in Finland.

^v^ Statistically significant difference at level p <.05 with vegetarians.

^l^ Statistically significant difference at level p <.05 with the low-RPM group.

^h^ Statistically significant difference at level p <.05 with the high-RPM group.

Appendix 4. Percentages of food choice motives, BMI, and lifestyle factors and adjusted mean and 95% confidence intervals (CI)^a^ for alcohol consumption (g/day) in the year 2017 in the groups of vegetarians (self-defined), low red and processed meat (RPM) consumption, or high RPM consumption.

|  |  | Vegetarians (n=98) | Low-RPM^b^ group (n=799) | High-RPM^c^ group  (n=888) |
| --- | --- | --- | --- | --- |
|  | n | % | % | % |
| Food choice motives: Important that food… |  |  |  |  |
| is high in meat | 1751 | 1.0^l,h^ | 13.8^v,h^ | 62.1^v,l^ |
| is high in FV | 1754 | 99.0^l,h^ | 90.6^v,h^ | 78.4^v,l^ |
| is low in fat | 1753 | 60.2 | 69.0^h^ | 50.3^l^ |
| is high in fiber | 1752 | 84.5^h^ | 84.2^h^ | 64.5^v,l^ |
| is low in salt | 1758 | 64.3^l^ | 76.0^v,h^ | 59.5^l^ |
| is low in carbohydrates | 1749 | 30.6 | 28.4^h^ | 23.2^l^ |
| contains no additives | 1753 | 80.6^h^ | 75.8^h^ | 59.0^v,l^ |
| comforts when sad or stressed | 1757 | 27.6^l,h^ | 15.5^v^ | 15.6^v^ |
| Possibility to eat in workplace/school canteen | 1762 | 43.2 | 31.4 | 35.3 |
| Eats lunch in workplace/school canteen | 1774 | 23.7^l^ | 13.8^v,h^ | 18.5^l^ |
| BMI <25 | 1759 | 50.0^h^ | 44.4^h^ | 25.6^v,l^ |
| Leisure-time PA: inactive | 1770 | 21.6 | 22.4 | 25.1 |
| Commuting PA: inactive | 1095 | 30.3^h^ | 37.3^h^ | 63.6^v,l^ |
| Work-related PA: inactive | 1750 | 60.0 | 68.3^h^ | 55.7^l^ |
| Smoking regularly | 1764 | 14.3 | 12.9^h^ | 20.5^l^ |
|  |  |  |  |  |
|  | n | Mean (95% CI) | Mean (95% CI) | Mean (95% CI) |
| Alcohol consumption^d †^ | 1785 | 3 (2-3)^h^ | 3 (2-3)^h^ | 5 (5-6)^v,l^ |

BMI, Body mass index; PA, Physical activity; RPM, Red and processed meat

^a^ Geometric mean and 95% CIs for LG10 transformed and back-transformed alcohol consumption variable, which was analyzed with analyses of covariance (Bonferroni corrections), adjusted for daily energy intake. All other variables were analyzed unadjusted (Chi-square test).

^b^ The lowest red and processed meat consumption quintile in the year 2017: cutoff point 54 g/day, excluding vegetarians.

^c^ The highest red and processed meat consumption quintile in the year 2017: cutoff point 160 g/day.

^d^ Measured as ethanol g/day.

^v^ Statistically significant difference at level p <.05 with vegetarians.

^l^ Statistically significant difference at level p <.05 with the low-RPM group.

^h^ Statistically significant difference at level p <.05 with the high-RPM group.

^†^ Gender interaction: men in the high-RPM group consumed more alcohol than those in the low-RPM group, no differences between groups among women.

Appendix 5. Percentages of food choice motives, BMI, and lifestyle factors and adjusted mean and 95% confidence intervals (CI)^a^ for alcohol consumption (g/day) in the year 2017 in the groups of vegetarians (cutoff point < 50 g/day of RPM, poultry, or fish), low red and processed meat (RPM) consumption, or high RPM consumption.

|  |  | Vegetarians (n=60) | Low-RPM^b^ group (n=828) | High-RPM^c^ group  (n=888) |
| --- | --- | --- | --- | --- |
|  | n | % | % | % |
| Food choice motives: Important that food… |  |  |  |  |
| is high in meat | 1742 | 0.0^l,h^ | 13.3^v,h^ | 62.1^v,l^ |
| is high in FV | 1745 | 100.0^l,h^ | 90.9^v,h^ | 78.4^v,l^ |
| is low in fat | 1744 | 58.3 | 68.6^h^ | 50.3^l^ |
| is high in fiber | 1752 | 84.5^h^ | 84.2^h^ | 64.5^v,l^ |
| is low in salt | 1749 | 63.3 | 75.6^h^ | 59.5^l^ |
| is low in carbohydrates | 1740 | 21.7 | 29.0^h^ | 23.2^l^ |
| contains no additives | 1744 | 80.0^h^ | 76.2^h^ | 59.0^v,l^ |
| comforts when sad or stressed | 1748 | 33.3^l,h^ | 15.7^v^ | 15.6^v^ |
| Possibility to eat in workplace/school canteen | 1753 | 41.4 | 32.1 | 35.3 |
| Eats lunch in workplace/school canteen | 1765 | 25.4^l^ | 14.0^v,h^ | 18.5^l^ |
| BMI <25 | 1750 | 53.4^h^ | 44.4^h^ | 25.6^v,l^ |
| Leisure-time PA: inactive | 1761 | 25.4 | 22.2 | 25.1 |
| Commuting PA: inactive | 1094 | 23.7^h^ | 37.1^h^ | 63.6^v,l^ |
| Work-related PA: inactive | 1741 | 64.9 | 67.5^h^ | 55.7^l^ |
| Smoking regularly | 1755 | 15.0 | 12.9^h^ | 20.5^l^ |
|  |  |  |  |  |
|  | n | Mean (95% CI) | Mean (95% CI) | Mean (95% CI) |
| Alcohol consumption^d †^ | 1776 | 2 (2-3)^h^ | 3 (2-3)^h^ | 5 (5-6)^v,l^ |

BMI, Body mass index; PA, Physical activity; RPM, Red and processed meat

^a^ Geometric mean and 95% CIs for LG10 transformed and back-transformed alcohol consumption variable, which was analyzed with analyses of covariance (Bonferroni corrections), adjusted for daily energy intake. All other variables were analyzed unadjusted (Chi-square test).

^b^ The lowest red and processed meat consumption quintile in the year 2017: cutoff point 54 g/day, excluding vegetarians.

^c^ The highest red and processed meat consumption quintile in the year 2017: cutoff point 160 g/day.

^d^ Measured as ethanol g/day.

^v^ Statistically significant difference at level p <.05 with vegetarians.

^l^ Statistically significant difference at level p <.05 with the low-RPM group.

^h^ Statistically significant difference at level p <.05 with the high-RPM group.

^†^ Gender interaction: men in the high-RPM group consumed more alcohol than those in the low-RPM group, no differences between groups among women.

Appendix 6. Adjusted means and 95% confidence intervals (CI)^a^ for consumption (g/day) of selected foods in the year 2017 in the groups of vegetarians, low red and processed meat (RPM) consumption, or high RPM consumption.

|  |  | Vegetarians (n=80) | Low-RPM^b^ group (n=812) | High-RPM^c^ group (n=888) |
| --- | --- | --- | --- | --- |
|  | n | Mean (95% CI) | Mean (95% CI) | Mean (95% CI) |
| RPM | 1690 | 11 (10-13)^l,h^ | 34 (33-36)^v,h^ | 195 (187-203)^v,l^ |
| Poultry | 1690 | 2 (1-3)^l,h^ | 23 (21-26)^v^ | 28 (26-31)^v^ |
| Fish | 1690 | 12 (9-14)^l,h^ | 40 (37-43)^v^ | 40 (37-42)^v^ |
| Eggs | 1690 | 18 (15-21)^h^ | 22 (21-24)^h^ | 30 (28-32)^v,l^ |
| Liquid dairy products | 1690 | 298 (248-357)^l^ | 394 (367-422)^v,h^ | 311 (291-333)^l^ |
| Cheese | 1690 | 39 (33-48) | 42 (39-45)^h^ | 34 (32-37)^l^ |
| Butter and butter-based fat spreads | 1690 | 6 (5-8)^h^ | 7 (7-8)^h^ | 9 (9-10)^v,l^ |
| Vegetable margarine and oil | 1690 | 15 (13-19) | 16 (15-17) | 15 (14-16) |
| FV | 1690 | 533 (468-608)^l,h^ | 401 (381-422)^v,h^ | 348 (331-366)^v,l^ |
| Legumes | 1690 | 37 (31-44)^l,h^ | 10 (9-11)^v^ | 10 (9-11)^v^ |
| Nuts and seeds | 1690 | 8 (6-10)^l,h^ | 5 (5-6)^v,h^ | 2 (2-3)^v,l^ |
| Cereal products | 1690 | 124 (113-136)^h^ | 122 (117-126)^h^ | 105 (101-109)^v,l^ |
| Rye^e^ | 1689 | 32 (26-42) | 31 (28-34)^h^ | 25 (23-27)^l^ |
| Sweets and chocolate | 1690 | 12 (10-15)^l^ | 9 (8-10)^v^ | 10 (9-11) |
| Sugary beverages | 1674 | 30 (22-43) | 24 (21-27)^h^ | 35 (31-40)^l^ |

FV, Fruit and vegetables; RPM, Red and processed meat

^a^ Geometric means and 95% CIs for LG10 transformed and back-transformed food consumption variables, which were analyzed with analyses of covariance (Bonferroni corrections), adjusted for daily energy intake, gender, age, education level and relative household income.

^b^ The lowest red and processed meat consumption quintile in the year 2017: cutoff point 54 g/day, excluding vegetarians.

^c^ The highest red and processed meat consumption quintile in the year 2017: cutoff point 160 g/day.

^e^ Rye is the most important determinant of whole grain intake in Finland.

^v^ Statistically significant difference at level p <.05 with vegetarians.

^l^ Statistically significant difference at level p <.05 with the low-RPM group.

^h^ Statistically significant difference at level p <.05 with the high-RPM group.
